# Arousal State-Dependence of Interactions Between Short- and Long-Term Auditory Novelty Responses in Human Subjects

**DOI:** 10.3389/fnhum.2021.737230

**Published:** 2021-10-01

**Authors:** Kirill V. Nourski, Mitchell Steinschneider, Ariane E. Rhone, Rashmi N. Mueller, Hiroto Kawasaki, Matthew I. Banks

**Affiliations:** ^1^Human Brain Research Laboratory, Department of Neurosurgery, The University of Iowa, Iowa City, IA, United States; ^2^Iowa Neuroscience Institute, The University of Iowa, Iowa City, IA, United States; ^3^Department of Neurology, Albert Einstein College of Medicine, Bronx, NY, United States; ^4^Department of Neuroscience, Albert Einstein College of Medicine, Bronx, NY, United States; ^5^Department of Anesthesia, The University of Iowa, Iowa City, IA, United States; ^6^Department of Anesthesiology, University of Wisconsin School of Medicine and Public Health, Madison, WI, United States; ^7^Department of Neuroscience, University of Wisconsin School of Medicine and Public Health, Madison, WI, United States

**Keywords:** auditory cortex, consciousness, general anesthesia, high gamma, iEEG, local/global deviant, predictive coding, propofol

## Abstract

In everyday life, predictable sensory stimuli are generally not ecologically informative. By contrast, novel or unexpected stimuli signal ecologically salient changes in the environment. This idea forms the basis of the predictive coding hypothesis: efficient sensory encoding minimizes neural activity associated with predictable backgrounds and emphasizes detection of changes in the environment. In real life, the brain must resolve multiple unexpected sensory events occurring over different time scales. The local/global deviant experimental paradigm examines auditory predictive coding over multiple time scales. For short-term novelty [hundreds of milliseconds; local deviance (LD)], sequences of identical sounds (/xxxxx/) are interspersed with sequences that contain deviants (/xxxxy/). Long-term novelty [several seconds; global deviance (GD)] is created using either (a) frequent /xxxxx/ and infrequent /xxxxy/ sequences, or (b) frequent /xxxxy/ and infrequent /xxxxx/ sequences. In scenario (a), there is both an LD and a GD effect (LDGD, “double surprise”). In (b), the global deviant is a local standard, i.e., sequence of identical sounds (LSGD). Cortical responses reflecting LD and GD originate in different brain areas, have a different time course, and are differentially sensitive to general anesthesia. Neural processes underlying LD and GD have been shown to interact, reflecting overlapping networks subserving the detection of novel auditory stimuli. This study examined these interactions using intracranial electroencephalography in neurosurgical patients. Subjects performed a GD target detection task before and during induction of anesthesia with propofol. Recordings were made from the auditory cortex, surrounding auditory-related and prefrontal cortex in awake, sedated, and unresponsive states. High gamma activity was used to measure the neural basis of local-by-global novelty interactions. Positive interaction was defined as a greater response to the double surprise LDGD condition compared to LSGD. Negative interaction was defined as a weaker response to LDGD. Positive interaction was more frequent than negative interaction and was primarily found in auditory cortex. Negative interaction typically occurred in prefrontal cortex and was more sensitive to general anesthesia. Temporo-parietal auditory-related areas exhibited both types of interaction. These interactions may have relevance in a clinical setting as biomarkers of conscious perception in the assessment of depth of anesthesia and disorders of consciousness.

## Introduction

In everyday life, sensory stimuli that are predictable are not very ecologically informative. Accordingly, neural activity elicited by such stimuli is dampened (reviewed in Nelken, 2014; Pérez-González and Malmierca, 2014). Unexpected stimuli stand out against the background of predictable stimuli. The *novelty* of the unexpected stimuli represents changes in the environment that may be ecologically salient. These unexpected sounds elicit larger neural responses in auditory processing networks compared to those elicited by the background (reviewed in Grimm and Escera, 2012).

The considerations noted above form the foundation for the predictive coding hypothesis for sensory processing. Expectations based on past sensory events generate feedback predictions within higher order cortical regions. Prediction signals are transmitted back to sensory cortices, resulting in diminished responses to the predicted stimuli (Mumford, 1992; Bastos et al., 2012). When sensory inputs violate these predictions, feedforward error signals are carried via ascending sensory pathways to higher order areas, and the dynamic model of the environment is updated. Predictive coding leads to metabolically efficient sensory processing, wherein resources are preserved and allocated to identify potentially important new information associated with changes in the environment.

Predictive coding in the auditory domain can be studied by presenting a background of frequent, predictable sounds (“*standards*”) and introducing infrequent, unpredictable sounds (“*deviants*”) against this background. Deviant stimuli are expected to elicit enhanced neural responses compared to those evoked by the standard stimuli. The difference between the two neural responses constitutes a *deviance effect*.

**FIGURE 1:**
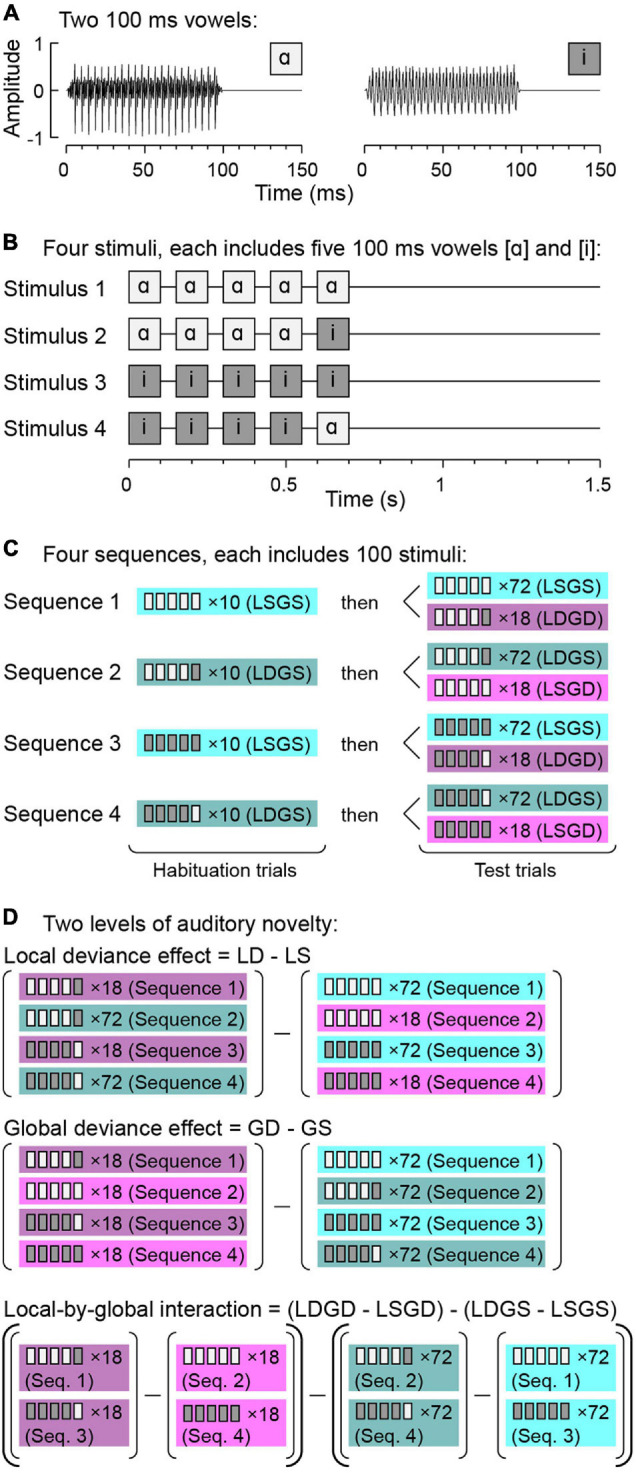
Local/global deviant (LGD) experimental paradigm. **(A)** Waveforms of the two vowels/ɑ/and/i/used to construct the experimental stimuli. **(B)** Schematic of the four experimental stimuli. **(C)** Stimulus sequences. **(D)** Comparisons between responses to specific stimulus types to characterize LD and GD effects and L × G interactions. Note that in subject R413, a slightly modified paradigm was used, where each sequence included 110 stimuli. The 10-trial habituation sequence was followed by 80 GS and 20 GD test trials in this subject. GD, global deviant; GS, global standard; LD, local deviant; LS, local standard. Modified from Nourski et al. (2018a).

In real-life situations, the brain does not process one prediction violation at a time. Instead, it must resolve layers of novel sensory events that occur over multiple time scales. In the auditory domain, the local/global deviant (LGD) paradigm (Bekinschtein et al., 2009) is a useful experimental tool to examine predictive coding mechanisms over two distinct time scales. In this paradigm, short-term novelty occurs over hundreds of milliseconds and is exemplified by presenting repetitive sounds, such as the vowel /ɑ/, and infrequently introducing a different sound, e.g., the vowel /i/ (Figures 1A,B). This short-term novelty is termed local deviance (LD).

The LGD paradigm also allows for investigation of novelty over longer time scales. For example, within a block of stimuli, repetition of a sequence of five identical vowels (e.g., /ɑɑɑɑɑ/) can be paired with occasional presentation of a sequence wherein the final vowel is replaced with another (e.g., /ɑɑɑɑi/). This leads to both the short-term novelty (LD) effect and a deviance effect over a longer time scale that is based on the change of the overall pattern of the five-vowel sequences, termed global deviance (GD).

In this example, there is both an LD and a GD effect when five identical vowels are replaced by an occasional sequence of five vowels with the last one different from the first four (“*double surprise*”). GD can also occur when the frequent sequence contains a local deviant, e.g., /ɑɑɑɑi/, and is occasionally replaced by a quintuple of five identical stimuli /ɑɑɑɑɑ/. Here, GD is not associated with LD, but instead is represented by a globally unexpected local standard.

Local deviance and GD effects can be measured using non-invasive methods such as electroencephalography (EEG) and magnetoencephalography (MEG) as differences between responses to standard and deviant stimuli (Bekinschtein et al., 2009; Recasens et al., 2014a,b). Results of source analysis of these responses suggest that different brain regions encode auditory novelty with distinct temporal profiles of neural activation (Recasens et al., 2014a,b). While EEG and MEG provide the necessary temporal resolution to identify neural activity associated with LD and GD, their spatial resolution is insufficient to resolve detailed patterns of activity within the auditory cortical hierarchy (Bekinschtein et al., 2009; Wacongne et al., 2011; Strauss et al., 2015).

Intracranial electroencephalography (iEEG) provides both the high spatial and temporal resolution needed to identify the neural correlates of novelty detection. Studies using iEEG have refined results of non-invasive studies by demonstrating that auditory novelty detection in an LGD paradigm engages multiple cortical regions at distinct time scales (King et al., 2013; El Karoui et al., 2015; Nourski et al., 2018a). The LD effect is associated with feedforward information flow from core (primary) auditory cortex to non-core auditory and auditory-related regions. By contrast, the GD effect appears to originate in posterior superior temporal gyrus (STG) and surrounding auditory-related areas, with subsequent propagation forward to prefrontal cortex and backward to core auditory cortex (Nourski et al., 2018a).

Recent non-invasive studies found evidence for interactions between LD and GD effects, suggesting that these two forms of deviance detection are not fully independent modes of auditory novelty processing (Shirazibeheshti et al., 2018; Kompus et al., 2020; Witon et al., 2020). *Local-by-global* (L × G) *interactions* can be measured by comparing responses to four stimulus conditions: LSGS, LSGD, LDGS and LDGD. Here, L and G denote local and global time scale, and S and D denote standard and deviant stimuli, respectively. Positive interaction is defined as a greater response to the double surprise LDGD condition compared to LSGD. Negative interaction is defined as a weaker response to the double surprise condition relative to LSGD.

Local-by-global interactions have been hypothesized to represent information flow between cortical networks that subserve short- and long-term novelty detection (Witon et al., 2020). Non-invasive studies have shown that neural responses to GD stimuli can be enhanced when these stimuli include LD (Wacongne et al., 2011; Shirazibeheshti et al., 2018). It is hypothesized that this increased response is based on the presence of a feedforward error signal provided by LD. Likewise, in the LSGD condition, the absence of this feedforward LD error signal can be expected to yield a diminished response to the LSGD stimulus.

A key consideration of auditory novelty detection is its modulation by arousal state. Within the predictive coding framework, the ongoing comparison of predictions and sensory observations is a fundamental feature of conscious sensory processing. Anesthetic-induced sedation and loss of consciousness (LOC) disrupt auditory predictive coding (Uhrig et al., 2016; Nourski et al., 2018b; Sanders et al., 2021). During anesthesia induced by propofol, LD effects are preserved within the auditory cortex when the subjects are unconscious, while GD effects are suppressed when subjects are sedated but still conscious (Nourski et al., 2018b).

The present work is the first iEEG study to investigate L × G interactions using the LGD paradigm. The goals of the study were four-fold: (1) Clarify the timing of positive and negative L × G interactions; (2) Identify the brain structures where these interactions occur; (3) Examine how these interactions are modulated during induction of general anesthesia with propofol; and (4) Differentiate attention- and task-related phenomena from those due to changes in arousal state.

These goals were addressed by using an active behavioral task which provided several advantages over a passive-listening setting. In a passive paradigm, absence of L × G interactions might simply be a function of inattention to the sound stimuli. Prevalence of high gamma GD effects is greater in an active paradigm compared to passive listening (Nourski et al., 2021b). Thus, it can be expected that L × G interactions would also be more prominent in an active task. Further, presence or loss of behavioral responses can serve as an additional criterion for defining the state of arousal. Finally, relating physiology and behavior helps identify neural activity contributing to task performance as opposed to less relevant neurophysiologic responses.

Cortical activity was measured in the high gamma iEEG band (70–150 Hz). High gamma is a surrogate of action potential firing in small neuronal populations. It provides a finer-grain spatial resolution compared to scalp EEG and intracranially recorded averaged evoked potentials (Steinschneider et al., 2008; Crone et al., 2011). In the present study, the gradual induction of general anesthesia allowed for a critically important comparison between sedated and unconscious states. Findings pertaining to sedation and unconsciousness may have translational relevance for the assessment of other altered states of arousal including sleep, delirium, and coma.

## Materials and Methods

### Subjects

Study subjects were seven adult neurosurgical patients (three female, four male, age 21–59 years old, median age 30 years old) with medically refractory epilepsy. The patients had been implanted with intracranial electrodes to identify resectable seizure foci. Subjects’ age, sex, electrode coverage, and seizure focus data are presented in Table 1. All subjects were native English speakers; all except one were right-handed and had left language dominance as determined by Wada tests (subject R413 was left-handed and right hemisphere-dominant).

**TABLE 1:** Subject demographics and electrode coverage.

Subject^1^	Age	Sex^2^	Number of recording sites per ROI							Seizure focus
			Auditory cortex			Auditory-related	Prefrontal	Other	Total	
			HGPM	STP	STG					
**R369**	30	M	8	15	17	79	39	54	212	R medial temporal
**L372**	34	M	6	12	25	51	34	49	177	L temporal pole
**R376**	48	F	7	10	18	76	30	52	193	R medial temporal
**R394**	24	M	8	2	0	6	2	7	25	R medial temporal
**R399**	22	F	3	6	21	46	47	60	183	R temporal
**L400**	59	F	4	7	3	25	54	65	158	L medial temporal
**R413**	21	M	8	12	25	81	45	52	223	R medial temporal
**Total number of recording sites**			44	64	109	364	251	339	1171	

*^1^Letter prefix of the subject code denotes the side of electrode implantation over auditory cortex and the side of seizure focus (L = left; R = right).*

*^2^F = female; M = male.*

All subjects underwent audiometric evaluation before the study, and none was found to have hearing deficits or word recognition scores sufficient to affect the findings presented in this study. Cognitive function, as determined by standard neuropsychological assessments, was in the average range in all subjects. Subject R394 had previously undergone a resection of a cavernoma in the anterior medial temporal lobe. The resection had spared cortex corresponding to all the brain regions of interests (ROIs) (see below) except for planum polare (PP). This subject had normal hearing and cognitive abilities and thus was included in the study.

The subjects were tapered off their antiepileptic drugs during the chronic monitoring and had their medication regimens reinstated to varying degrees at the end of the monitoring period, prior to the electrode removal and seizure focus resection surgery.

### Stimuli and Procedure

Experiments were conducted in the operating room immediately prior to and during induction of general anesthesia for electrode removal and seizure focus resection surgery. The experiments were part of a series of studies on auditory novelty detection and resting state connectivity across task conditions and arousal states (Nourski et al., 2018a,b, 2021b,c; Banks et al., 2020). Auditory stimuli were quintuples of vowels /ɑ/ and /i/, presented in an LGD paradigm (Bekinschtein et al., 2009; Nourski et al., 2018a; Figure 1). The vowels were edited (duration 100 ms) from the steady-state vocalic portions of consonant-vowel stimuli /hɑd/ and /hid/, spoken by a female (fundamental frequency 232 and 233 Hz, respectively) (Hillenbrand et al., 1995). The vowels were normalized to the same root-mean-square amplitude and gated with 5 ms on/off ramps (Figure 1A). On each trial, four identical vowels, separated by 50 ms intervals, were presented, followed by either the same or different fifth vowel (Figure 1B). This within-quintuple difference constituted short term (local) deviance: /ɑɑɑɑɑ/ and /iiiii/ were LS stimuli, while /ɑɑɑɑi/ and /iiiiɑ/ were LD.

The stimuli were presented in blocks of four sequences, with the order of the sequences randomized across blocks (Figure 1C). In all subjects except R413, each sequence began with a recorded instruction that defined the task and the target (GD) stimulus to the subject, e.g., for Sequence 1: “This time, press the button when you hear this sound: /ɑɑɑɑi/. Once again, press the button when you hear this sound: /ɑɑɑɑi/.” The instruction was followed by a habituation sequence of 10 trials that established the GS condition (e.g., /ɑɑɑɑɑ/ for Sequence 1), and then by 72 GS and 18 GD test trials, presented in a pseudorandom order. The difference in presentation frequency constituted the long term (global) deviance, and the identity of the GD stimulus changed across the four sequences within each block (Figure 1D). Note that the infrequent (GD) trials could have either five identical vowels (LSGD) or a different fifth vowel (LDGD). Likewise, the frequent (GS) trials either had the fifth vowel same or different as the first four (LSGS and LSGD, respectively). The intertrial interval varied within a Gaussian distribution (onset-to-onset mean 1500 ms, standard deviation 10 ms) to reduce heterodyning in the recordings secondary to the 60 Hz power line noise.

In subject R413, a simplified protocol was used, where instead of a recorded instruction, the task was explained beforehand to the subject by the researcher as follows: “Press the button every time you hear the sound sequence change.” In this subject, each 10-trial habituation sequence was followed by 80 GS and 20 GD test trials. The duration of each experimental block was 11 min in all subjects.

Stimuli were presented by a TDT RZ2 processor (Tucker-Davis Technologies, Alachua, FL, United States) and delivered at a comfortable level (60–65 dB SPL) diotically via insert earphones (ER4B, Etymotic Research) enclosed in custom-fit earmolds. The subjects were instructed to operate the response button with the hand ipsilateral to the hemisphere from which recordings were made. This was done to minimize contributions to recorded neural responses from activity reflecting motor planning and execution, and somatosensory responses associated with the button press.

**FIGURE 2:**
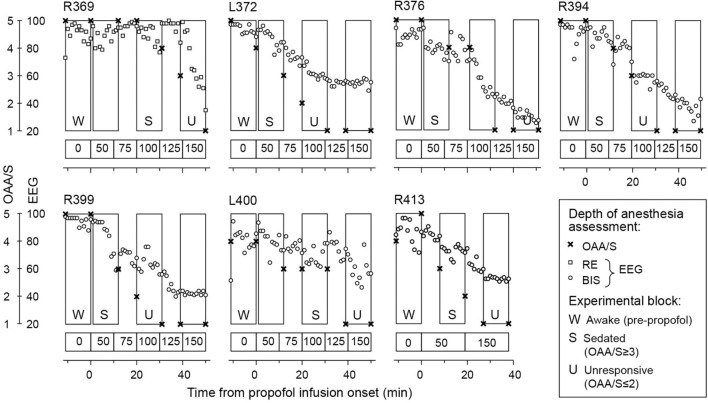
Induction of general anesthesia. Observer’s Assessment of Alertness/Sedation (OAA/S) scores (crosses) and electroencephalographic (EEG) sedation indices are plotted as functions of time. EEG-based sedation indices were response entropy [RE] in subject R369 and bispectral index (BIS) in all other subjects (open squares and circles, respectively). Rectangles denote 11-min LGD experimental blocks; letter labels indicate the three arousal states (W: awake, S: sedated, U: unresponsive). Propofol infusion rates are shown underneath each plot.

Each experiment included three or four 11-min blocks. The first block was presented immediately before administration of propofol. Following the completion of the first block, infusion of propofol was initiated at a rate of 50 μg/kg/min (Alaris pump, BD, Maplewood, MO, United States). Propofol was the sole sedative drug administered to the patients during the experimental period. The time course of induction of sedation followed by general anesthesia is shown for each subject in Figure 2. In all subjects except R413, the rate of infusion was increased every 10 min by 25 μg/kg/min, following the approach previously used by Nourski et al. (2017,2018b,2021b) and Banks et al. (2020). The duration of the infusion was 50 min with a maximum rate of 150 μg/kg/min. Three auditory stimuli blocks were presented during the 50 min. In subject R413, a simplified protocol was used, wherein the rate of infusion was 50 μg/kg/min for 20 min, followed by an increase to 150 μg/kg/min for another 20 min. An auditory stimulus block was presented during the final 11 min of each of these two 20-min periods. The infusions were supervised by an attending anesthesiologist using standard respiratory and hemodynamic monitoring. None of the infusions had to be interrupted or terminated for the patients’ safety.

The depth of sedation was evaluated before and after each block using the Observer’s Assessment of Alertness/Sedation (OAA/S) scale, the gold standard in assessing alertness in the clinical setting (Chernik et al., 1990). Responsiveness (calling the subject’s name), speech (asking the subject to repeat the sentence, “The quick brown fox jumps over the lazy dog”), facial expression (the degree of facial relaxation), and eyes (the subject’s ability to focus and ptosis) were all assessed and scored on a scale from 1 to 5. The composite OAA/S score, ranging from 5 (“alert”) to 1 (“deep sleep”), was defined as the lowest level indicated by any of the four assessment categories.

For the purposes of analyses, three arousal states were defined in each subject: awake (W; before administration of propofol), sedated (S) and unresponsive (U). The letter “W” is used throughout the manuscript instead of “A” for “awake” to avoid the possibility of the abbreviated “A” being interpreted as “Anesthesia.” The transition from OAA/S = 3 (“responsive to loud or repeated command”) to OAA/S = 2 (“unresponsive in the absence of mild prodding or shaking”) (Chernik et al., 1990) was used as the threshold between sedation and LOC. LOC was thus approximated as the loss of responsiveness (Vanluchene et al., 2004; Nourski et al., 2018b; Banks et al., 2020). The depth of sedation was additionally assessed using EEG parameters: response entropy (RE) (E-ENTROPY module; Datex-Ohmeda, Madison, WI, United States) (Viertiö-Oja et al., 2004) in subject R369 and bispectral index (BIS) (BIS Complete 4-Channel Monitor; Medtronic, Fridley, MN, United States) (Gan et al., 1997) in all other subjects. The EEG parameters were recorded continuously throughout each experiment and were manually logged on a minute-by-minute basis.

### Recording

Intracranial electrophysiological recordings were made using depth and subdural electrodes (Ad-Tech Medical, Oak Creek, WI, United States) implanted to identify potentially resectable seizure foci (Nagahama et al., 2018b). Electrode implantation, recording, and iEEG data analysis have been previously described in detail (Nourski and Howard, 2015). Depth electrode arrays (8–12 cylindrical macro contacts spaced 5 mm apart) targeting the superior temporal plane (STP) including Heschl’s gyrus, were stereotactically implanted along the anterolateral-to-posteromedial axis of the gyrus. Depth electrodes which targeted insular cortex provided additional coverage of posteromedial portion of Heschl’s gyrus (HGPM), planum temporale (PT), and PP. This configuration was clinically warranted, as it bracketed the suspected temporal lobe seizure foci for their accurate assessment (Nagahama et al., 2018a). Subdural strip and grid electrode arrays consisted of platinum-iridium disc contacts (2.3 mm exposed diameter, 5–10 mm contact-to-contact distance) embedded in a silicone membrane. They were implanted over lateral and ventral cerebral surfaces. A subgaleal electrode was used as a reference.

Reconstruction of the anatomical locations of implanted electrode contacts in individual subjects and their mapping onto a standardized set of coordinates was performed using FreeSurfer image analysis suite (Version 5.3; Martinos Center for Biomedical Imaging, Harvard, MA, United States) and in-house software. Subjects underwent T1-weighted whole-brain structural 3T magnetic resonance imaging (MRI) scans (resolution 1.0 mm) before electrode implantation and MRI and computerized tomography (CT) scans (resolution 1.0 mm) after implantation. Locations of the electrode contacts were obtained from post-implantation MRI and CT scans and projected onto pre-operative MRI scans using non-linear three-dimensional thin-plate spline morphing and intraoperative photography. The locations were then transformed into standard Montreal Neurological Institute (MNI) coordinates using linear co-registration to the MNI152 T1 average brain, as implemented in FMRIB Software library (Version 5.0; FMRIB Analysis Group, Oxford, United Kingdom). For recording sites in the left hemisphere, MNI *x*-axis coordinates (*x*_*MNI*_) were multiplied by (−1) to map them onto the right-hemisphere common space.

**FIGURE 3:**
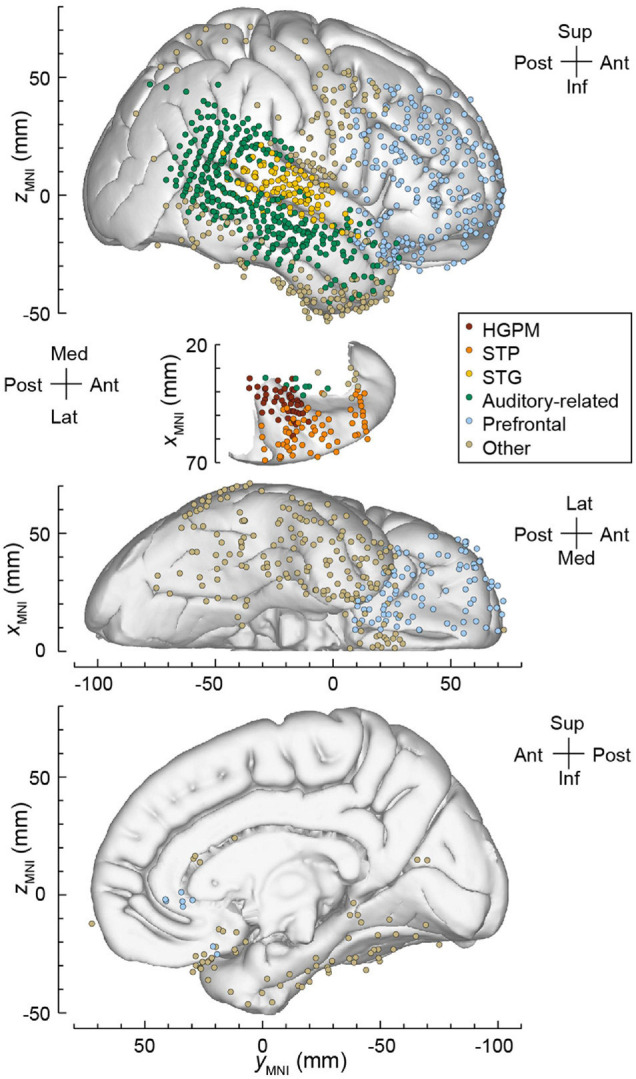
Electrode coverage in all seven subjects. Locations of recording sites, color-coded according to the ROI, are plotted in MNI coordinate space and projected onto the right hemisphere of the Freesurfer average template brain for spatial reference. Left hemisphere MNI *x*-axis coordinates (*x*_*MNI*_) were multiplied by –1 to map them onto the right-hemisphere common space. Projection is shown on the lateral, top-down (STP), ventral and mesial views **(top** to **bottom)**. Recording sites over orbital, transverse frontopolar, inferior temporal gyrus and temporal pole are shown in both the lateral and the ventral view. Sites in fusiform, lingual, parahippocampal gyrus and gyrus rectus are shown in both the ventral and medial view. Sites in the hippocampus (*n* = 13), amygdala (*n* = 12), frontal operculum (*n* = 5), parietal operculum (*n* = 3), substantia innominata (*n* = 5), putamen (*n* = 1), and uncus (*n* = 1) are not shown. HGPM, posteromedial portion of Heschl’s gyrus; STP, superior temporal plane; STG, superior temporal gyrus.

The locations of recording sites were projected onto the right lateral hemispheric surface, STP, ventral and mesial views of the FreeSurfer average template brain (Figure 3). The electrode coverage in all subjects is summarized in Table 1. The following ROIs were identified, spanning the hierarchy of auditory cortical processing (a modification of the scheme used previously in Nourski et al., 2018a,b, 2021a,c; Banks et al., 2020):

(1) Core auditory cortex in the posteromedial portion of Heschl’s gyrus (HGPM; *n* = 44 sites).
(2) Non-core auditory cortex in the STP (*n* = 64), including the anterolateral portion of Heschl’s gyrus (HGAL; *n* = 25), PT (*n* = 21), and PP (*n* = 18).
(3) Non-core auditory cortex on the STG (*n* = 109), including its posterior (*n* = 72) and middle (*n* = 37) portions.
(4) Temporo-parietal auditory-related cortex (*n* = 364), including the posterior insula (*n* = 8), anterior STG (*n* = 17), superior temporal sulcus (upper bank, STSU: *n* = 12; lower bank, STSL: *n* = 19), and middle temporal (MTG; *n* = 187), supramarginal (SMG; *n* = 65), and angular (AG; *n* = 56) gyri.
(5) Prefrontal cortex (*n* = 251), including the inferior (IFG; *n* = 55), middle (MFG; *n* = 80), and superior (SFG; *n* = 15) frontal gyri, orbital (OG; *n* = 76) and transverse frontopolar gyri (TFG; *n* = 19), and anterior cingulate cortex (*n* = 6).

An additional 339 recording sites provided coverage of other brain areas, including the inferior temporal gyrus (ITG) (*n* = 62), temporal pole (*n* = 58), precentral (*n* = 44), postcentral (*n* = 30), parahippocampal (*n* = 21), fusiform gyrus (*n* = 20), gyrus rectus (*n* = 20), premotor cortex (*n* = 14), hippocampus (*n* = 13), amygdala (*n* = 12), anterior insula (*n* = 8), middle occipital gyrus (*n* = 6), superior parietal lobule (*n* = 6), frontal operculum (*n* = 5), substantia innominata (*n* = 5), cingulate gyrus (*n* = 4), parietal operculum (*n* = 3), lingual gyrus (*n* = 2), inferior occipital gyrus (*n* = 2), cuneus (*n* = 2), putamen (*n* = 1), and uncus (*n* = 1).

Assignment of recording sites to ROIs was based on anatomical reconstructions of electrode locations in each subject. For subdural arrays, it was informed by automatic parcelation of cortical gyri as implemented in the FreeSurfer image analysis suite (Destrieux et al., 2010, 2017). Heschl’s gyrus was subdivided into HGPM and HGAL. The boundary between the two was defined physiologically based on the presence of phase-locked responses to click train stimuli and short-latency components in averaged evoked potentials. These features are characteristic of HGPM and are absent in HGAL (Brugge et al., 2009). STG was subdivided into posterior and middle non-core auditory cortex portions, and auditory-related anterior portion using the transverse temporal sulcus and ascending ramus of the Sylvian fissure as macroanatomical boundaries. For depth electrodes, ROI assignment was informed by MRI sections along sagittal, coronal, and axial planes. The insula was subdivided into the auditory-related posterior portion and anterior insular cortex (Zhang et al., 2019). Within cingulate gyrus, anterior cingulate cortex (as identified by automatic parcelation in FreeSurfer) was considered a prefrontal area and thus examined separately from the rest of cingulate cortex. Recording sites identified as seizure onset zones or those characterized by excessive noise, as well as depth electrode contacts located outside cortical gray matter, were excluded from analyses and thus are not listed in Table 1.

Behavioral (button presses) and iEEG data were recorded using the TDT RZ2 processor; iEEG data were amplified, filtered (0.7–800 Hz bandpass, 12 dB/octave rolloff) and digitized at a sampling rate of 2034.5 Hz.

### Data Analysis

Analysis of data was performed using software written in MATLAB R2020a (MathWorks, Natick, MA, United States). Behavioral performance in the target detection task was characterized as accuracy (hit rate, i.e., the percentage of correctly detected target stimuli), sensitivity (*d’* = *Z*_*hit*_-*Z*_*false alarm*_, where *Z* is the inverse of the cumulative distribution function of the normal distribution) and reaction times (RTs). These metrics were computed separately for LDGD and LSGD trials in each awake and sedated block. Only button presses that occurred between the onset of the 5th vowel and the onset of the 1st vowel of the following trial were considered hits. Button presses that overlapped with the next non-target trial were considered false alarms. The behavioral results thus likely somewhat underestimated target detection rates and biased the RTs toward faster responses. Hit rates and *d*’ values were compared between LDGD and LSGD trials across subjects using one-tailed Wilcoxon signed rank tests. RTs were compared between LDGD and LSGD trials using Wilcoxon rank sum tests. *P*-values were corrected for multiple comparisons using the false discovery rate (FDR) approach (Benjamini and Hochberg, 1995).

Analysis of iEEG data focused on power in high gamma band (70–150 Hz). Data were downsampled to 1000 Hz, denoised using demodulated band transform approach (Kovach and Gander, 2016) and bandpass-filtered (300th order finite impulse response filter, 70–150 Hz passband). Voltage deflections of the high gamma band-filtered signal that exceeded five standard deviations from the across-block mean for each recording site were considered artifacts. Trials that contained such deflections were excluded from further analysis. The high gamma signal was then squared and smoothed using a 50 ms running average window to obtain high gamma power. Power (μV^2^) was used rather than voltage or dB-transformed event-related band power because response waveforms must be non-negative signals for the sign of the L × G interaction to be interpretable.

Responses were averaged across LSGS, LDGS, LSGD, and LDGD test trials separately (see Figure 1D, bottom row). L × G interactions were calculated as the difference of the differences of high gamma responses to the four stimulus types, i.e.,:


L×G=(LDGD -LSGD) - (LDGS -LSGS)

Local-by-global interaction waveforms were baseline-corrected by subtracting the mean value over the 600 ms prior to the onset of the 5th vowel.

The statistical significance of L × G interactions was examined within the time interval between 0 and 800 ms following the onset of the 5th vowel. Significance was established using a non-parametric cluster-based permutation test (Maris and Oostenveld, 2007; Nourski et al., 2018a). The test statistic was based on grouping adjacent time points that exhibited L × G interactions. The cluster statistic for each recording site and experimental block was obtained by first computing *t*-values across all time points. At each time point, *t*-values were compared to a threshold value (the 1st percentile tail of the two-tailed *T*-distribution). Clusters were defined as consecutive time points for which the *t*-values exceeded the threshold, and the cluster-level statistic was computed as the sum of the *t*-values within each cluster. The *p*-values were calculated using permutation tests in which 10,000 random trial partitions were shuffled with respect to the four trial labels. Cluster-level statistics were calculated, and the largest cluster-level statistic was identified for each partition. Monte Carlo *p*-values were calculated for each cluster based on the 10,000-sample distribution set of the test statistics. Interactions were considered significant at *p* < 0.05. Recording sites with at least one significant positive or negative L × G interaction cluster were considered as exhibiting the corresponding interaction type.

The spatial distribution of L × D interactions across the lateral hemispheric surface and the STP was visualized by plotting locations of sites characterized by significant positive or negative interactions in the MNI coordinate space and projecting them onto the right hemisphere of the FreeSurfer average template brain. ROIs were characterized in terms of the prevalence of positive and negative L × G interactions in each of the three arousal states. Prevalence was defined as the percentage of sites exhibiting a significant interaction in each arousal state. The onset latency of L × G interactions was defined as the beginning of the first significant cluster and calculated separately for positive and negative interactions. Onset latencies of positive interactions in the awake state were compared between HGPM, STP, STG, and auditory-related cortex using the Kruskal–Wallis test. For positive interaction, comparison of onset latencies in these ROIs between awake and sedated states was done using the Wilcoxon rank sum test. Likewise, for negative interaction, comparison of onset latencies between auditory-related and prefrontal cortex in the awake state was done using the Wilcoxon rank sum test. The overall time course of positive and negative interactions was visualized by plotting *T*-scores, averaged across sites that exhibited significant interactions, as functions of time after the 5th vowel onset.

## Results

### Task Performance

**FIGURE 4:**
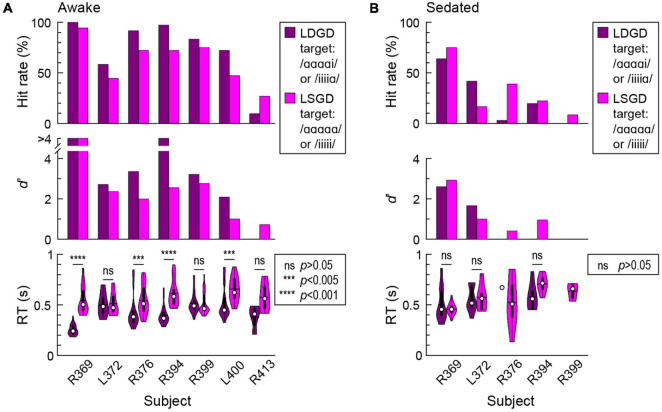
Global deviance (GD) target detection task performance in the awake **(A)** and sedated **(B)** states. Summary of data from seven subjects. Hit rates (% correctly detected target stimuli), sensitivity (*d*’) and RTs are plotted in the top, middle, and bottom panels, respectively. In the sedated state, subject R399 did not have correct hit responses to LDGD targets, and subjects L400 and R413 did not have correct hit responses to either LSGD or LDGD targets.

All seven subjects performed the GD target detection task to varying degrees, as measured by hit rates, *d’* and RTs, during awake and sedated experimental blocks. The “double surprise” LDGD condition typically provided an advantage for the performance of the GD detection task compared to the LSGD target condition (Figure 4). In the awake state, the LDGD condition was associated with higher hit rates in six out of seven subjects (Figure 4A, top panel), though this improvement did not reach significance (*p* = 0.055). Sensitivity (*d’*) for LDGD target trials was higher than for LSGD trials in five subjects (Figure 4A, middle panel), and the improvement was statistically significant (*p* = 0.016). Finally, the LDGD condition was associated with significantly faster behavioral responses in four subjects (R369: ΔRT = 263 ms, *p* < 0.0001; R376: ΔRT = 130 ms, *p* = 0.00192; R394: ΔRT = 216 ms, *p* < 0.0001; L400: ΔRT = 174 ms, *p* = 0.00249) (Figure 4A, bottom panel). Across all hit trials and subjects, the grand median RTs for LDGD and LSGD 420 and 516 ms, respectively.

Sedation with sub-hypnotic doses of propofol led to a deterioration of task performance (Figure 4B). Subjects R376 and R399 only had one and zero correct hit responses to LDGD targets, respectively. Subjects L400 and L413 only had false alarm responses to both types of GD targets in the sedated state. None of the remaining three subjects exhibited a significant difference in RTs between LSGD and LDGD target trials. Sedation with propofol thus appeared to decrease the advantageous behavioral effect of “double surprise” provided by the LDGD condition in the awake state.

### Electrophysiological Signatures of Local-by-Global Interactions

**FIGURE 5:**
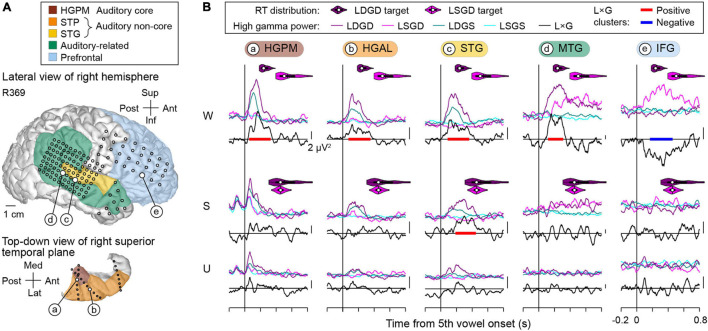
L × G interactions during the induction of general anesthesia in a representative subject with right hemisphere electrode coverage (R369). **(A)** Lateral view of the right hemispheric surface and top-down view of the STP depicting electrode coverage. Colors represent different ROIs, circles represent recording sites. Larger white circles denote the locations of five representative recording sites (a–e). **(B)** High gamma responses to the final vowel of the LGD quintuplet stimulus recorded from the exemplary sites (a–e, left to right) and L × G interactions in awake, sedated, and unresponsive states (W, S, U; top to bottom). Across-trial average high gamma power envelopes are shown separately for the four stimulus conditions (LSGS, LDGS, LSGD, and LDGD; cyan, teal, magenta, and purple, respectively). Black lines denote L × G interaction time course, baseline-corrected by subtracting mean value over the 600 ms prior to the onset of the 5th vowel. Vertical scale bars correspond to 2 μV^2^. Significant (*p* < 0.05) positive and negative L × G interaction clusters are shown as red and blue bars, respectively. RT distributions for LSGD and LDGD target stimuli are shown as magenta and purple violin plots, respectively. In each violin plot, a white circle denotes the median, a vertical line denotes the mean, a bar denotes *Q*1 and *Q*3, and whiskers show the range of lower and higher adjacent values (i.e., values within 1.5 interquartile ranges below *Q*1 or above *Q*3, respectively).

The use of subdural and depth arrays allowed for a comprehensive assessment of responses from multiple cortical regions comprising the auditory processing hierarchy. This assessment is exemplified by data from subject R369, who displayed the best task performance of all subjects (Figure 5). Coverage of the right hemispheric convexity by subdural electrode arrays is depicted along with a top-down view of the STP which illustrates the placement of depth arrays (Figure 5A). High gamma responses and L × G interactions at selected sites during awake (W), sedated (S), and unresponsive (U) states are shown in Figure 5B. As the main effects of LD and GD have been reported elsewhere (Nourski et al., 2018a,b), analyses presented below will focus solely on L × G interactions.

In subject R369, the awake state featured a *positive* L × G interaction within core auditory cortex (HGPM), surrounding auditory cortical areas (HGAL, lateral STG) and in auditory-related cortex (MTG) (Figure 5B, top row). Significant positive interaction (denoted by red bars in Figure 5B) emerged within 100 ms and peaked between 200 and 300 ms after the onset of the 5th vowel. By contrast, the IFG site was characterized by a *negative* L × G interaction, wherein LSGD stimuli elicited larger responses than LDGD beyond LD effect (blue bar in Figure 5B). This interaction developed later than the positive L × G interaction, emerging at around 200 ms after the 5th vowel onset in this example. The onset of both types of L × G interactions preceded the subjects’ behavioral responses to the respective trials (see violin plots in Figure 5B). Sedation with propofol was associated with attenuation of L × G interactions. In the example shown in Figure 5B (middle row), the STG site was the only site that maintained a significant positive L × G interaction, while the negative interaction in the IFG site was absent. L × G interactions were abolished in the unresponsive state (see Figure 5B, bottom row).

**FIGURE 6:**
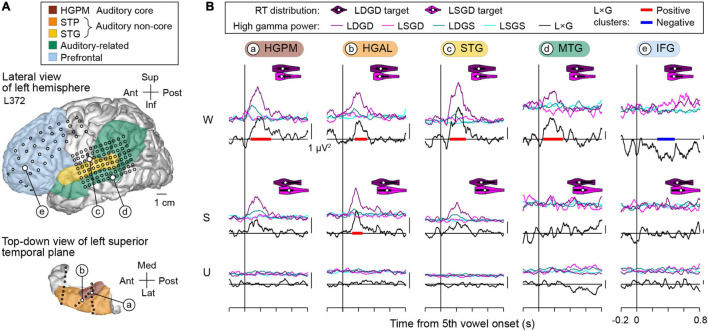
L × G interactions during induction of general anesthesia in a representative subject with left hemisphere electrode coverage (L372). See caption of Figure 5 for detail.

Positive and negative L × G interactions were present in both hemispheres, as exemplified by data obtained from the left hemisphere in subject L372 (Figure 6). This subject exhibited below-average hit rates in the task and no significant RT difference between LSGD and LDGD. In the awake state, positive L × G interaction occurred in core, non-core auditory, and auditory-related cortex, and negative L × G interaction was identified in the IFG. As seen in the previous example, L × G interactions were strongly modulated by propofol. In the sedated and unresponsive state, there were no significant interactions except for a positive L × G interaction at the HGAL site in the sedated state.

The two examples above demonstrate positive and negative L × G interactions in both language-dominant and non-dominant hemisphere and in both above- and below-average task performers. Positive interaction preceded negative interaction and occurred at earlier stages within the cortical processing hierarchy. At all examined stages of cortical auditory processing, sedation with propofol strongly diminished these physiologic interactions, which were further attenuated in the unresponsive state.

### Spatial Distribution and Time Course of Local-by-Global Interactions

**FIGURE 7:**
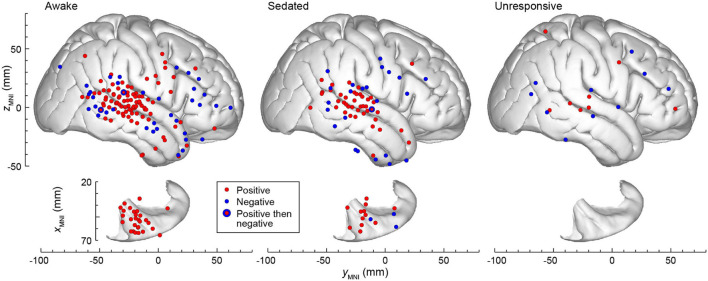
The topography of L × G interactions across states of arousal. A summary of data from seven subjects, plotted in the MNI coordinate space and projected onto the right hemisphere of the FreeSurfer average template brain for spatial reference. Top-down views of the right superior temporal plane are plotted underneath side views of the right lateral hemispheric convexity and aligned along the *y*_*MNI*_ axis. Sites that exhibited positive and negative L × G interactions are depicted by red and blue symbols, respectively. Note that some sites in ventral prefrontal cortex (IFG and OG) appear over anterior STG when projected onto the template brain. Sites in the STSU and STSL are projected onto the lateral hemispheric convexity and thus appear to be over either STG or MTG.

The spatial distribution of L × G interactions across all subjects in the three states of arousal is summarized in Figure 7. The data were plotted in the MNI coordinate space and projected onto the right hemisphere of the FreeSurfer average template brain to allow for pooling of data from multiple subjects. Marked differences were present in the spatial distribution of positive and negative interactions. Only positive interaction was identified in the STP in the awake state. The auditory cortex on the lateral STG generally exhibited positive interaction whereas the surrounding auditory-related cortex exhibited both positive and negative interactions. Negative interaction was more common than positive in prefrontal cortex. In the three sites that featured both positive and negative interactions (a posterior STG and an MTG site in the awake state, and another posterior STG site in the sedated state), positive interaction preceded negative one. Increasing sedation by the administration of escalating doses of the propofol infusion led to a progressive decrease in the number of sites exhibiting L × G interactions. Eventually, when the unresponsive state was achieved, very few sites with significant interactions remained in the studied brain regions.

The distributions of L × G effects were examined with respect to responses to the vowel stimuli, LD and GD effects, as reported for this subject cohort in previous studies (Figure 3B in Nourski et al., 2021c and Figure 4 in Nourski et al., 2018b). In the awake state, sites that were responsive to the vowel stimuli yet exhibited no significant L × G interactions of either type, clustered in HGPM and PT. With sedation, there was an increased incidence of sites throughout the STP (except PP) and on the lateral STG. When the subjects became unresponsive, the prevalence of both types of L × G interaction markedly diminished compared to prevalence of responses to vowel stimuli both in the STP and on the lateral STG. Sites that exhibited a significant LD effect without a significant L × G interaction were present in all three studied arousal states, and their distribution (STP and lateral STG) was relatively consistent across the three states. Finally, sites that exhibited a significant GD effect without a significant L × G interaction mostly clustered in posterior auditory-related and prefrontal areas. With sedation, only a few such sites remained, reflecting a sharp decline in the prevalence of GD effect with sedation. In the unresponsive state, there were no sites that exhibited a significant GD effect and no L × G interaction.

**FIGURE 8:**
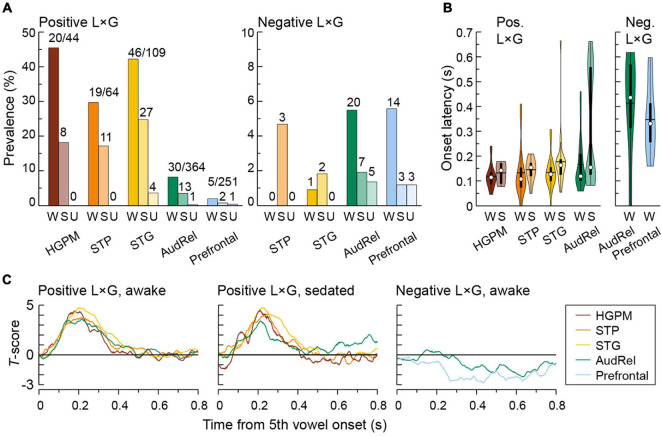
Regional distribution and time course of L × G interactions. Summary of data from seven subjects. Colors represent ROIs, differently shaded bars and symbols represent measurements made in awake (W), sedated (S), and unresponsive (U) state (dark, medium, and light bar shading, respectively). **(A)** Percentages of sites with significant positive and negative L × G interactions (left and right panel, respectively). Numbers above bars indicate numbers of sites with significant L × G interactions. Note different *y*-scales in the left and right panel. **(B)** Onset latencies of positive and negative L × G interactions (left and right panel, respectively). In each violin plot, a white circle denotes the median, a horizontal line denotes the mean, a bar denotes *Q*1 and *Q*3, and whiskers show the range of lower and higher adjacent values (i.e., values within 1.5 interquartile ranges below *Q*1 or above *Q*3, respectively). The latency distributions for negative L × G interaction are only shown for the awake state, as very few sites exhibited this interaction in the sedated state (7 and 3 sites in auditory-related and prefrontal cortex, respectively), making statistical inferences impractical. **(C)** Time course of L × G interactions. *T*-scores averaged across sites that exhibited significant interactions are plotted as functions of time after the 5th vowel onset. In panels **(B,C)**, positive L × G interaction in prefrontal cortex and negative L × G interaction in canonical auditory cortex (HGPM, STP, and STG) are not shown due to their paucity in the respective regions.

The distribution of L × G interactions across ROIs, their onset latency and overall time course are examined in Figure 8. Overall, negative interaction was seen far less frequently than positive, as reflected in the different *y*-scales in Figure 8A. In the awake state, the prevalence of positive interaction was the highest in the canonical auditory cortex with prevalence in HGPM, STP, and STG of 45.5, 29.7, and 42.2%, respectively (Figure 8A, left panel). An intermediate response pattern with both positive and negative interactions was observed in the auditory-related cortex. The prevalence of negative interaction was greatest in the prefrontal and auditory-related cortex (5.58 and 5.49%, respectively) (Figure 8A, right panel). Sedation and loss of responsiveness were associated with a progressive decline in the prevalence of both types of interactions.

In the awake state, onset latencies of positive interaction were comparable between HGPM, STP, STG, and auditory-related cortex (median values 115, 108, 128, and 120 ms, respectively; *p* = 0.210, Kruskal–Wallis test) (Figure 8B, left panel). There was a significant increase in the onset latency of the positive L × G interaction between the awake and sedated states (median latencies 117 and 159 ms, respectively; *p* = 0.000893, Wilcoxon rank sum test) within the auditory and auditory-related cortex. Onset latencies of negative interaction were much longer than of positive interaction, with median values in auditory-related and prefrontal cortex of 441 and 335 ms, respectively. However, the difference between onset latencies in the two ROIs did not reach statistical significance in this limited data set (*p* = 0.100, Wilcoxon rank sum test) (Figure 8B, right panel). As even fewer sites exhibited this interaction in the sedated state (7 and 3 sites in auditory-related and prefrontal cortex, respectively), statistical inferences regarding latency were not feasible in this case.

The overall time course of positive and negative interactions is depicted in Figure 8C. The time course of both types of interactions was similar across the canonical auditory and auditory-related cortex in the awake and sedated states. This paralleled the similar onset latencies of positive interaction in these regions. The positive interaction peaked at around 200 ms and extended to around 400 ms after the onset of the final vowel. Negative interaction in auditory-related and prefrontal cortex had a slower time course.

Outside of core auditory cortex, there was variability in the prevalence of L × G interactions across subdivisions within each ROI (Table 2). Within the STP, PT exhibited the greatest prevalence of positive L × G interaction in the awake state (52.4%); negative interaction was not observed at all. By contrast, L × G interactions were virtually absent in PP. There was a progressive decrease in the prevalence of positive interaction from the posterior to middle to anterior STG (51.4, 24.3, and 5.88%, respectively). Negative interaction was very infrequent in all three subdivisions.

**TABLE 2:** Numbers and percentages of sites with significant positive and negative L × G interactions across arousal states.

ROI	*n*_*total*_	Positive L × G interaction						Negative L × G interaction					
		W		S		U		W		S		U	
		*n*	%	*n*	%	*n*	%	*n*	%	*n*	%	*n*	%
**HGPM**	**44**	**20**	**45.5**	**8**	**18.2**	**0**	**0**	**0**	**0**	**0**	**0**	**0**	**0**
**STP**	**64**	**19**	**29.7**	**11**	**17.2**	**0**	**0**	**0**	**0**	**3**	**4.69**	**0**	**0**
HGAL	25	7	28	5	20	0	0	0	0	1	4	0	0
PT	21	11	52.4	5	23.8	0	0	0	0	0	0	0	0
PP	18	1	5.56	1	5.56	0	0	0	0	2	11.1	0	0
**STG**	**109**	**46**	**42.2**	**27**	**24.8**	**4**	**3.67**	**1**	**0.972**	**2**	**1.83**	**0**	**0**
Posterior STG	72	37	51.4	19	26.4	2	2.78	1	1.39	2	3.78	0	0
Middle STG	37	9	24.3	8	21.6	2	5.41	0	0	0	0	0	0
**Auditory-related**	**364**	**30**	**8.24**	**13**	**3.57**	**1**	**0.275**	**20**	**5.49**	**7**	**1.92**	**5**	**1.37**
Anterior STG	17	1	5.88	0	0	0	0	1	5.88	0	0	0	0
STSU	12	4	33.3	0	0	0	0	3	25	0	0	0	0
STSL	19	4	21.1	1	5.26	0	0	1	5.26	0	0	0	0
MTG	187	9	4.81	4	2.14	1	0.535	9	4.81	3	1.6	2	1.07
SMG	65	6	9.23	4	6.15	0	0	3	4.62	3	4.62	1	1.54
AG	56	5	8.93	3	5.36	0	0	3	5.36	1	1.79	2	3.57
**Prefrontal**	**251**	**5**	**1.99**	**2**	**0.797**	**1**	**0.398**	**14**	**5.58**	**3**	**1.2**	**3**	**1.2**
IFG	55	2	3.64	0	0	0	0	6	10.9	2	3.63	1	1.82
MFG	80	1	1.25	1	1.25	0	0	4	5	1	1.25	2	2.5
SFG	15	0	0	0	0	0	0	0	0	0	0	0	0
OG	76	1	1.32	1	1.32	1	1.32	3	3.95	0	0	0	0
TFG	19	0	0	0	0	0	0	0	0	0	0	0	0
**Other**	**339**	**21**	**6.19**	**3**	**0.885**	**3**	**0.885**	**7**	**2.06**	**19**	**5.6**	**2**	**0.59**
Inferior temporal g.	62	3	4.84	1	1.61	0	0	0	0	5	8.06	1	1.61
Temporal pole	58	2	3.45	1	1.72	0	0	0	0	2	3.45	0	0
Precentral g.	44	8	18.2	0	0	0	0	1	2.27	3	6.82	0	0
Postcentral g.	30	1	3.33	0	0	0	0	1	3.33	2	6.67	0	0
Parahippocampal g.	21	0	0	0	0	0	0	1	4.76	1	4.76	0	0
Fusiform g.	20	1	5	0	0	1	5	0	0	1	5	0	0
G. rectus	20	0	0	0	0	0	0	0	0	3	15	0	0
Premotor cortex	14	1	7.14	0	0	1	7.14	0	0	0	0	1	7.14
Hippocampus	13	0	0	0	0	0	0	1	7.69	0	0	0	0
Amygdala	12	0	0	0	0	0	0	0	0	0	0	0	0

*ROI subdivisions that had electrode coverage of <10 sites are not shown.*

*AG, angular gyrus; g., gyrus; HGAL, anterolateral Heschl’s gyrus; HGPM, posteromedial Heschl’s gyrus; IFG, inferior frontal gyrus; L × G, local-by-global; MFG, middle frontal gyrus; MTG, middle temporal gyrus; OG, orbital gyrus; PP, planum polare; PT, planum temporale; ROI, region of interest; S, sedated; SFG, superior frontal gyrus; SMG, supramarginal gyrus; STSL, lower bank of the superior temporal sulcus; STSU, upper bank of the superior temporal sulcus; STG, superior temporal gyrus; TFG, transverse frontopolar gyrus; U, unresponsive; W, awake.*

A marked difference in the prevalence of positive and negative interactions occurred outside of auditory cortex. The prevalence of positive and negative interactions in the awake state was similar in the three subdivisions of auditory-related cortex with extensive electrode coverage (MTG, SMG, and AG). This increase in prevalence of negative interaction culminated in the IFG. Of 55 sites in the IFG, where 2 (3.64%) sites showed positive interaction while 6 (10.9%, a two-fold increase compared to overall prevalence within prefrontal cortex) exhibited negative interaction. None of the 34 recording sites in the SFG and TFG had either type of interaction. The highest percentage of interactions in other areas examined was in the precentral gyrus. Here, 8 out of 44 sites (18.2%) showed a positive interaction, while only one site displayed negative interaction in the awake state.

**FIGURE 9:**
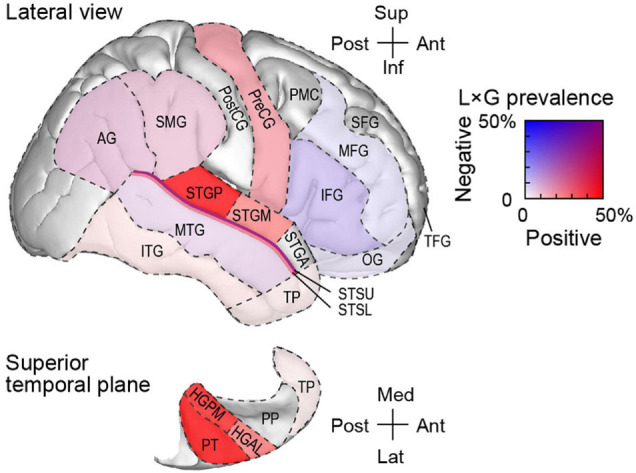
Schematic of regional distribution of L × G interactions in the awake state. ROIs are color-coded according to prevalence of positive and negative interactions. For ROIs where only a single site exhibited a significant interaction (PP, STGA, PostCG, and PMC), prevalence estimates are not shown. See text and Table 2 for details. AG, angular gyrus; HGAL, anterolateral Heschl’s gyrus; HGPM, posteromedial Heschl’s gyrus; IFG, inferior frontal gyrus; L × G, local-by-global; MFG, middle frontal gyrus; MTG, middle temporal gyrus; OG, orbital gyrus; PMC, premotor cortex; PP, planum polare; PreCG, precentral gyrus; PostCG, postcentral gyrus; PT, planum temporale; ROI, region of interest; SFG, superior frontal gyrus; SMG, supramarginal gyrus; STSL, STSU, lower and upper bank of the superior temporal sulcus, respectively; STGA, STGM, STGP, posterior, middle, and anterior superior temporal gyrus, respectively; TFG, transverse frontopolar gyrus; TP, temporal pole.

The regional distribution of L × G interactions presented in detail in Table 2 is graphically summarized in Figure 9. Here, ROIs are color-coded based on the prevalence of positive and negative interactions in the awake state. Caution must be exercised when extrapolating the prevalence of these interactions in each ROI. First, it should not be assumed that interactions are homogenously distributed throughout each ROI, especially outside canonical auditory cortex (cf. Figure 7). Second, the prevalence was calculated based on limited sample sizes in several of the ROIs (cf. Table 2). Thus, this graphical summary warrants conservative interpretation. Still, it is evident that positive L × G interaction primarily occurred in the auditory cortex on the STP, lateral STG (except rostral areas PP and STGA), and precentral gyrus. By contrast, negative interaction primarily occurred within the IFG, and became progressively less prevalent at more dorsal and rostral prefrontal areas. Finally, multiple auditory-related ROIs exhibited both types of interaction.

## Discussion

### Summary of Findings

The present study extends previous findings of auditory novelty processing (Nourski et al., 2018a,b, 2021b,c) by specifically examining neural responses that reflect interactions of LD and GD in the LGD paradigm. Identifying where and when these interactions occur provides insight into how the brain manages to simultaneously analyze multiple levels of novelty, as encountered in typical sound environments. Changes in these interactions may be relevant for understanding altered auditory novelty detection in states of reduced arousal. These considerations elevate L × G interactions from a purely experimental observation to a biologically relevant phenomenon.

The main finding of this study is that different brain regions are associated with positive and negative L × G interactions (see Figure 9). Positive interaction occurs in the canonical auditory cortex and, to a lesser degree, in the precentral gyrus (areas shaded in red in Figure 9). Negative interaction primarily occurs in the prefrontal cortex, more specifically in IFG (shaded blue in Figure 9) and, to a lesser extent, MFG and OG. Auditory-related areas are associated with both types of interaction (shaded purple in Figure 9). Behaviorally, GD is more salient when paired with the feedforward error signal associated with LD (“double surprise”). This is manifested as an enhancement in performance on the GD target detection task. By contrast, GD is less salient when where is no feedforward error signal. Paradoxically, the LSGD condition, which produces smaller responses in auditory cortex, can elicit larger responses in higher-order cortical regions particularly within prefrontal cortex. The physiologic profile for the LSGD combination is characterized by longer onset latencies and parallels the greater task difficulty as measured by lower hit rates and *d*’, and longer RTs.

### Relationship to the Literature

In the original report introducing the LGD paradigm, no interactions were observed between LD and GD effects as measured by event-related potentials (Bekinschtein et al., 2009). This negative result has been subsequently attributed due to a non-standard method used to measure the interactions (Shirazibeheshti et al., 2018). An additional factor could be the use of the event-related potential as a response metric instead of a rectified signal (e.g., EEG power) (cf. Witon et al., 2020). The current study provides direct evidence for positive and negative L × G interactions by measuring high gamma power in iEEG recordings. The focus on high gamma activity was motivated by its high spatial specificity (Crone et al., 2011) and its interpretation as a surrogate for action potential firing within neuronal populations (Steinschneider et al., 2008).

A theoretical framework that accounts for the interactive component of the LGD paradigm has been proposed by Witon et al. (2020). In this framework, three phases of auditory novelty processing are envisioned. The early phase (100–150 ms) is characterized by detection of LD in the auditory cortex and includes the pre-attentive component of stimulus-specific adaptation (SSA) (Ulanovsky et al., 2003; Fishman and Steinschneider, 2012). The late phase (400–600 ms) is characterized by conscious attention-dependent detection of GD that is carried out by higher-order areas such as the IFG (Nourski et al., 2018a). Finally, the intermediate phase (250–350 ms) is postulated to represent bidirectional information exchange between the auditory cortex and IFG that underlies L × G interactions (Witon et al., 2020). Positive interaction as measured by intracranially recorded high gamma activity emerges earlier than that detected by the scalp EEG study of Witon et al. (2020) but otherwise overlaps with the intermediate processing phase. This interaction localizes to multiple areas within the auditory cortex and extends into adjacent auditory-related areas.

The onset latencies of responses to sound tend to increase along the auditory hierarchy, with the shortest latencies being in the core auditory cortex in HGPM (Nourski et al., 2014). This progressive increase in latency has been interpreted to reflect feedforward information flow from lower to higher auditory cortical regions (Nourski et al., 2021a). LD effects follow this feedforward latency pattern (Nourski et al., 2018a). Interestingly, this sequential increase in latency was not observed when examining positive L × G interaction along the auditory cortical hierarchy. Onset latencies of this interaction were similar across the auditory and auditory-related cortex. The reasons for this similarity in latency are unclear. It may be necessary to examine effective connectivity patterns to address this issue.

This iEEG study confirms the existence of a negative L × G interaction within the inferior frontal cortex, as first demonstrated by Witon et al. (2020) using scalp-recorded EEG. In the current study using iEEG, negative interaction was also observed in other areas of prefrontal cortex (MFG, OG). Another novel finding of his study was the prominence of negative interaction in auditory-related cortex (see Table 2). This effect was widespread and occurred in areas strongly associated with canonical auditory cortex (e.g., STSU) as well as higher-order associative regions (e.g., AG).

Unfortunately, onset latency data were not adequate to address whether the origin of negative interaction was within the prefrontal cortex and if this interaction was then transmitted to the auditory-related cortex via feedback connections. The median and mean latencies were shorter in the prefrontal compared to auditory-related cortex. However, the overall distributions of onset latencies were not significantly different between the two ROIs (at *p* = 0.10). Given the relative paucity of negative interaction, this question will have to be addressed by a future study employing a larger cohort of subjects with comprehensive electrode coverage of the relevant cortical regions.

### Effects of Propofol-Induced Sedation and Unresponsiveness

The principal effect of propofol is the attenuation of L × G interactions, with a greater effect on negative interaction. This effect is consistent with the previously reported results obtained during recovery from propofol-induced sedation (Shirazibeheshti et al., 2018; Witon et al., 2020). The use of a novel slow induction protocol in the present study allowed for a comparison between the sedated and unresponsive states. Both positive and negative interactions were attenuated by propofol upon sedation and were essentially abolished upon LOC.

Previous work has shown loss of GD effects (measured by combining LSGD and LDGD trials) at subhypnotic doses of propofol when subjects were sedated, but still responsive (Nourski et al., 2018b). This study indicates that extension of the LGD paradigm into the clinical realm using scalp-recorded data could focus on the positive L × G interaction. By contrast, given the greater sensitivity of the negative interaction to subhypnotic doses of propofol, negative interaction would likely be of a more limited utility in assessing pathologic states of consciousness.

### Mechanisms of Novelty Detection and Local-by-Global Interactions Across the Auditory Processing Hierarchy

Local deviance effects measured in the LGD paradigm are closely related to mismatch negativity (MMN) (Näätänen and Alho, 1995). Two mechanisms have been proposed as contributing to MMN (and, by proxy, to the LD effect). These are (1) SSA, which refers to the attenuation of responses to the repetition of the same stimuli (Fishman, 2014); and (2) A higher-level process that reflects stored neuronal memory of acoustic patterns which have been established by repeated sounds (Näätänen et al., 2005). SSA is present in the ascending auditory pathways (Malmierca et al., 2009; Antunes et al., 2010; Richardson et al., 2013) and the primary auditory cortex (Ulanovsky et al., 2003; Farley et al., 2010; Fishman and Steinschneider, 2012). It can occur in the anesthetized state (Duque and Malmierca, 2015) and operates even when a single token stimulus precedes a subsequent token. To identify acoustic patterns made up of multiple tokens, deviance detection must occur over longer temporal intervals (Ulanovsky et al., 2004). The regions surrounding primary auditory cortex have been shown to operate over progressively longer temporal intervals and thus conform to this requirement (Sharpee et al., 2011).

The finding that LSGD stimuli elicited larger responses than to LDGD in higher-order brain areas, but not auditory cortex, was unexpected given that the fifth vowel is the same as the first four. It would be expected that SSA would lead to a diminished response to the fifth vowel in the LSGD condition. Therefore, the larger responses to LSGD stimuli must be based on additional mechanisms beyond SSA.

Global deviance effects result from integration of sensory inputs over longer temporal intervals than that required for LD detection. The mechanisms for GD detection likely engage broader cortical networks of auditory working memory and parallel that seen in the multiscale processing of human speech. For example, a study that examined processing of narrated stories at the word, sentence, and paragraph level identified brain regions associated with the processing of speech over these respective temporal scales (Lerner et al., 2011). There was a progressive activation of ever-higher level auditory and auditory-related cortical regions which paralleled the processing of speech at the three levels of increasing complexity. The highest degree of activation involved in processing at the paragraph level occurred in prefrontal and parietal networks. In a similar manner, GD effects also require integration of information over long temporal windows and engage prefrontal and parietal regions (Nourski et al., 2018a, 2021b). Outside the canonical auditory cortex, regions in the auditory processing hierarchy operate over the progressively longer time scales required to detect long-term novelty within sound patterns (Ulloa et al., 2008; Farbood et al., 2015).

### Caveats and Limitations

A key concern regarding iEEG studies carried out in neurosurgical patients with epilepsy is that the experimental subjects are not entirely representative of a healthy population. With regards to the present study, consistent effects were observed across subjects despite differences in seizure disorder histories, antiepileptic medication regimens, and the location of seizure foci. Importantly, the findings of the present study are comparable to results obtained previously in healthy subjects using the same experimental paradigm and similar analyses of non-invasive recordings (Shirazibeheshti et al., 2018; Witon et al., 2020).

The variability of the effects of propofol in individual subjects represents a caveat specific to this investigation. Although the time course of the induction of general anesthesia varied across subjects, the arousal states were not defined by a specific dose or plasma concentration of propofol. Instead, arousal states were defined using the OAA/S, which is considered the gold standard for assessing awareness in the clinical setting (Chernik et al., 1990; Vanluchene et al., 2004).

Finally, for several reasons, the nature of the study precluded formal assessment of possible relationships between task performance and the electrophysiological L × G interaction profiles. First, L × G interaction–the neural response metric considered in the present study–is defined as the difference of differences between averaged responses to the four types of stimuli, i.e., (LDGD−LSGD)−(LDGS−LSGS). This complicates identification of relationships between behavioral performance and this particular facet of neural activity on a single-trial level. The relatively small subject sample (seven participants) with variable electrode coverage and the overall relatively low prevalence of significant L × G interactions also limited our ability to directly assess the relationship between physiology and behavior. Continuing this experimental paradigm in additional subjects will be required to formally address this important question.

### Future Directions and Clinical Implications

Key future experiments will include examining LGD effects during sedation and unresponsiveness induced by different anesthetic drugs with different cellular mechanisms of action. In addition to the studies that use anesthetics to probe LGD effects and their interactions, future work will examine the systems-level mechanisms of LGD detection during stages of natural sleep. The translational relevance of this work will be enhanced by combining intracranial and scalp-recorded activity to relate changes in scalp-recorded potentials to their intracranial sources. This will be important to improve prognostic accuracy in patients with disorders of consciousness (e.g., delirium and coma) which are a major problem in current neurologic practice.

## Data Availability Statement

The raw data supporting the conclusions of this article will be made available by the authors, without undue reservation.

## Ethics Statement

The studies involving human participants were reviewed and approved by University of Iowa Institutional Review Board. The patients/participants provided their written informed consent to participate in this study.

## Author Contributions

KN, MS, and MB: conception and design of the work. KN, RM, HK, and MB: acquisition of data. KN, MS, AR, and MB: analysis and interpretation of data. KN and MS: drafting the work. AR, RM, HK, and MB: editing the work. All authors provided approval for publication of the content and agreed to be accountable for all aspects of the work in ensuring that questions related to the accuracy or integrity of any part of the work are appropriately investigated and resolved.

## Conflict of Interest

The authors declare that the research was conducted in the absence of any commercial or financial relationships that could be construed as a potential conflict of interest.

## Publisher’s Note

All claims expressed in this article are solely those of the authors and do not necessarily represent those of their affiliated organizations, or those of the publisher, the editors and the reviewers. Any product that may be evaluated in this article, or claim that may be made by its manufacturer, is not guaranteed or endorsed by the publisher.
